# Exploring user characteristics, motives, and expectations and the therapeutic alliance in the mental health conversational AI Clare®: a baseline study

**DOI:** 10.3389/fdgth.2025.1576135

**Published:** 2025-06-13

**Authors:** Lea Maria Schäfer, Tabea Krause, Stephan Köhler

**Affiliations:** 1 Department of Psychiatry and Neurosciences, Charité – Universitätsmedizin Berlin, Campus Mitte, Berlin, Germany; 2 clare&me GmbH, Berlin, Germany; 3 Department of Psychiatry, Psychotherapy and Psychosomatics, St Joseph Hospital Berlin Weissensee, Berlin, Germany

**Keywords:** artificial intelligence, conversational agent, AI voicebot, feasibility, AI-supported psychotherapy, clinical large language model, help-seeking motives

## Abstract

This study examined the characteristics, motives, expectations, and attitudes of users interested in artificial intelligence (AI) self-help provided by the bot Clare®, a conversational AI for mental health support, and explored the development of a working alliance. A cross-sectional survey of 527 English-speaking self-referred users revealed high levels of anxiety (69%), depression (59%), severe stress (32%), and loneliness (86%). The participants expressed positive attitudes toward digital mental health solutions, with key motives including avoiding embarrassment (36%) and concerns about appearance in face-to-face consultations (35%). Expectations focused on emotional support (35%) and expressing feelings (32%). A strong working alliance was established within 3–5 days (Working Alliance Inventory-Short Report, *M* = 3.76, SD = .72). These findings highlight the potential of conversational AI in providing accessible and stigma-free support, informing the design of human-centric AI in mental health. Future research should explore long-term user outcomes and clinical large language model integration with traditional mental health services.

## 1 Introduction

A major barrier to the implementation and scale-up of mental health services is the persistent global shortage of mental health workers, currently estimated at 1.18 million professionals (1, 2). This workforce gap significantly limits service availability, with approximately 85% of individuals with mental health disorders in low- and middle-income countries receiving no care (3). Additional barriers, such as stigma, limited access, and a preference for self-care, deter many from seeking help, while others face delays due to a shortage of mental health professionals, particularly in rural and low-income areas (4, 5). Therefore, self-care interventions that focus on mental health and wellbeing have shifted into focus (6). Meanwhile, artificial intelligence (AI) and machine learning technologies are rapidly advancing in mental healthcare, offering new opportunities for diagnosis and treatment (7–11). Conversational AI (CAI), powered by natural language processing (NLP) and machine learning (ML), shows promise in reaching large, underserved populations (12). CAI can provide psychological care to vulnerable groups, such as the elderly, adolescents, and those avoiding traditional treatments due to financial or social barriers, such as fear of stigmatization (13, 14). Existing industry-driven CAIs, such as Woebot® or Wysa®, targeting depression, anxiety, and stress (12, 15, 16), show promising results in terms of symptom reduction (16) or bonding experience (17).

While early CAIs, such as ELIZA, an early rule-based chatbot developed by computer scientist Joseph Weizenbaum in the 1960s, primarily used (18), newer systems such as Wysa®, Woebot®, and Clare® leverage large language models (LLMs) to enable more flexible and context-aware conversations (19). These models can respond to unanticipated user inputs and adapt dynamically during interactions (20). Most digital solutions for mental health have been struggling with low engagement and high dropout rates (21). Proactive CAI, combining machine learning and LLMs, has potential to allow for an autonomous and digital provision of evidence-based therapeutic interventions that may improve adherence (22). AI, including LLMs, generates flexible and context-dependent responses and thereby mimics therapeutic conversations (23).

Despite their potential, CAIs also present ethical and safety concerns, particularly regarding patient autonomy, trust, and the risk of over-reliance on technology (13, 24). Additionally, research in this field is still scarce and only a very small number of CAIs incorporating LLMs have been tested, such as the industry-based mental health chatbot, Youper, which is based on rule-based and generative AI (25). In particular, advancements in proactive CAI have shifted the focus to topics such as conversational safety, response appropriateness, and AI alignment [see (19, 26) for an extensive overview]. Users' opinions about AI self-help are critical for successful implementation; hence, a research-driven framing of CAI appears to be crucial. The success of these AI tools depends on managing user expectations and addressing these concerns effectively (14). Moreover, we have limited knowledge of the sociodemographics, needs, and preferences of users of conversational AI for self-help, including their age and gender distribution and their expectations and motivations for seeking AI-driven mental health support.

This study explores the demographics, psychological wellbeing, motives, and attitudes of Clare® users, a conversational agent integrating rule-based and generative AI. Further, we assess users’ motives for using and expectations of AI self-help, perceptions of online interventions in general, and bonding effects after initial interactions with Clare®. Notably, in this study, Clare® was implemented as a voice-based conversational agent, enabling spoken interactions rather than solely text-based communication. Our findings aim to inform research-driven expectation management and guide the responsible design of human-centric CAIs.

## 2 Materials and methods

### 2.1 Clare®—the intervention itself

#### 2.1.1 Onboarding

Once participation was confirmed, access to Clare® was granted. The participants initiated the onboarding process via a sign-up website. This included an initial onboarding call with Clare® to schedule future calls. Subsequently, the users could trigger on-demand calls with Clare® through a dedicated study website.

#### 2.1.2 CAI-delivered intervention

Clare®, developed by clare&me GmbH, is an AI mental health support agent with emotional intelligence, available as a chat and voice agent on mobile phones. It offers 24/7 anonymous access without installation, simulating human-like, empathetic conversations. Clare® provides content based on cognitive behavioral therapy (CBT), self-compassion, and mindfulness (27–29). In this study, Clare® was used as a voice-only conversational agent.

Clare® integrates CBT techniques, including Socratic dialogue, cognitive restructuring, and framing, using a rule-based model developed by clinical psychologists to match user input with relevant exercises (e.g., meditation for stress). Users can choose the interaction length and mode (verbal or chat). For example, in response to “I feel tired and stressed,” Clare® identifies issues (e.g., sleep difficulties or stress) and provides suitable exercises (e.g., sleep hygiene or stress management). Examples of the content in Clare® are shown in Table 2. The design of Clare® was guided by key ethical principles, including:

- Accountability and transparency: Clearly disclosing the AI's functionalities, establishing boundaries for interaction, and educating users to manage expectations and foster trust.
- Data security and privacy: Adhering to established privacy standards to safeguard user information.
- Human-centered ethics: Aligning with ethical frameworks, such as the American Psychiatric Association (APA) Ethics Code, and prioritizing a human-in-the-loop approach to ensure appropriate oversight in mental health contexts.

#### 2.1.3 Exposure to Clare®

Participants could freely determine their level of interaction with Clare®. However, to be included in the analysis for working alliance, users were required to have had at least one onboarding call (see Table 2 and Section 2.4). To participate in the 8-week study, users were required to complete four questionnaires: at baseline, 3–5 days later (t1b), in week 4 (t2), and in week 8 (t3), and to be exposed to Clare® at least once in each interval between the measurement points. Exposure was defined as a bot-delivered phone call and characterized by the extent of participant interaction, including call frequency and duration. Observed behavioral patterns will not be analyzed in depth in this study but will be examined in a future publication.

#### 2.1.4 Engagement metrics

Engagement was quantified using two metrics: the number of bot-delivered phone calls per week and the average duration of these automated calls. All the call sessions included in the engagement metrics were automated interactions between the users and the voice-based bot. Participants were considered active users if they completed at least one bot-delivered phone call (including the onboarding session) with Clare® within 1 week.

#### 2.1.5 Technological base of Clare®

Clare® is a CAI built on multiple fine-tuned LLMs, developed by clare&me GmbH using various open-source LLMs. Fine-tuning involved collaboration with conversation designers and psychologists to ensure the communication aligns with therapeutic ethical guidelines, focusing on relationship formation, transparency, and limitations. Clare® operates independently, accepting text and voice inputs, with voice transcriptions processed using NLP to extract key information about emotions and context. Clare® engages users to assess their mental state and suggests relevant exercises, offering alternatives if declined. Interaction duration typically ranges from 5 to 45 min, depending on the iterative process and exercise length.

#### 2.1.6 Safety system

Clare® employs a robust safety framework during onboarding and ongoing use, emphasizing automatic crisis detection. A custom moderation application programming interface (API) filters inputs and outputs, flagging inappropriate content before LLM processing. Key safety features include the following.

##### 2.1.6.1 Crisis intervention

If suicidality or severe distress is detected, users are directed to psychological support hotlines and blocked from further use to ensure safety.

##### 2.1.6.2 Conduct enforcement

Users are reminded to interact respectfully and maintain a civil tone.

The safety system ensures appropriate referrals in emergencies and enforces conduct guidelines. A conservative approach prioritizes overflagging, with human moderators reviewing flagged content to minimize false positives.

### 2.2 Study design and procedure

Here we report cross-sectional data from a longitudinal study set within a larger research frame assessing the feasibility and effectiveness of the voice and chatbot Clare® that is yet to be published. In the Clare® trial, self-report assessments were performed at baseline (t1), after initial contact (t1b), after 4 weeks (t2), and after 8 weeks (t3). Further, we report baseline data from assessment points t1 and t1b for working alliance [Working Alliance Inventory-Short Report (WAI-SR)] (Table 1).

**Table 1:** Measures and timing.

Days after baseline questionnaire	Assessment timeframe	Assessment instrument
0	Baseline (t1)	APOI; SWLS; PHQ-4; PHQ-D; UCLA; Mini-Spin Motives and expectations as list to vote
3–5	After 5–7 days (t1b)	WAI-SR
28	During (t2)	SWLS; PHQ-4; PHQ-D; UCLA; Mini-Spin; WAI-SR; mARM; UEQ
76	Post (t3)	APOI; SWLS; PHQ-4; PHQ-D; UCLA; Mini-Spin; WAI-SR; mARM; UEQ

APOI, Attitudes towards Psychological Online Interventions Questionnaire; SWLS, Satisfaction with Life Scale; PHQ-4, 4-item Patient Health Questionnaire; PHQ-D, 10-item Patient Health Questionnaire; UCLA, UCLA Loneliness Scale; Mini-SPIN, 3-item Social Phobia Inventory; WAI-SR, Working Alliance Inventory-Short Revised; mARM, mobile Agnew Relationship Measure; UEQ, User Experience Questionnaire; motives and expectations were on a dichotomous scale (yes/no).

#### 2.2.1 Sample recruitment and data collection

Participants were recruited through English-language advertisements on Facebook, Instagram, LinkedIn, and Google Ads, using language targeting based on users' browser or account settings. No demographic targeting was applied. Digital access and literacy were assessed via questions on internet and mobile phone access, and prior use of Clare® or similar mental health tools. After providing informed consent, participants began the baseline survey (t1). Eligible participants met the inclusion criteria, completed the first survey, and had at least one onboarding call and interaction with Clare®.

##### 2.2.1.1 Inclusion criteria

**Figure 1:**
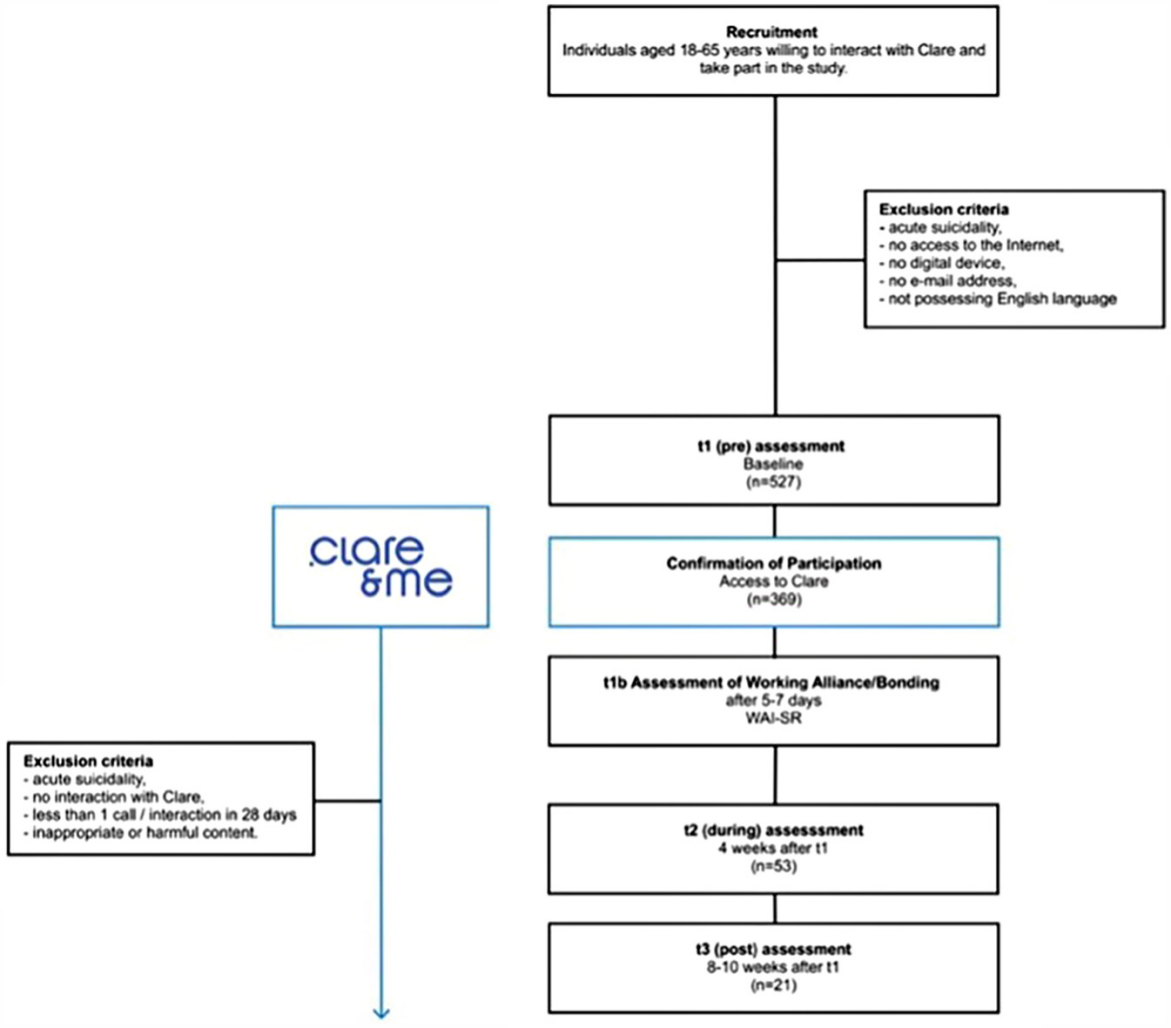
Flowchart. WAI-SR, Working Alliance Inventory-Short Revised. clare&me logo used with permission from https://www.clareandme.com/.

The participants were 18–65 years old, proficient in English, provided electronic consent and contact information, and had access to a mobile internet device. For the working alliance assessment, participants were required to schedule and complete an onboarding phone call with Clare® (approximately 3–6 min, see Table 2). Continued study participation over 8 weeks required completion of four questionnaires: a baseline questionnaire, a short questionnaire 3–5 days after onboarding with Clare® to assess the working alliance, and two follow-up questionnaires at 4-week intervals (t2, t3). In addition, the participants were required to engage with Clare® at least once between each measurement point. Behavioral engagement was defined as interactions between the users and Clare®, such as scheduling, receiving, and completing phone calls with Clare® (Figure 1).

**Table 2:** Overview of exemplary interactions with Clare®.

Dimensions	Content	Exercise name
Onboarding	Getting to know each other, a short questionnaire to collect general information about this person and their problems, users learn about the limits and background of AI self-help (transparency)	Suicide assessment, prioritization of problems and goals, miracle question
Resources-activation	Resources are activated, positive experiences are explored with the user and enforced	Resource-activation
Sleep pattern	Psychoeducation and introduction of a sleep ritual	Sleep hygiene
Automatic thoughts	Psychoeducation on rumination and worry, how to differentiate and understand automatic thoughts, dealing with worries and rumination, and practicing acceptance	Strategy of attention training, postpone worry, worry time technique, radical acceptance

##### 2.2.1.2 Exclusion criteria

Participants with acute suicidality, identified during onboarding by Clare®'s safety system, were excluded (see Section 2.1.6 for details). Participants who did not successfully onboard with Clare® were excluded from the working alliance analysis (t1b); individuals who did not fulfill the minimum behavioral engagement with Clare® were also excluded from this analysis.

### 2.3 Ethics approval

The study protocol was approved by the Ethics Committee of Campus Charité Mitte, Berlin, Germany (EA1/109/22).

### 2.4 Assessment instruments

At baseline (t1), 527 participants were assessed after confirming their eligibility (Figure 1). The online survey, administered via SoSciSurvey between 20 November 2023 and 21 April 2024, included 57 questions plus demographic data to capture participant characteristics and behaviors (Table 1). Although several additional variables were collected as part of the broader exploratory study (Table 1), only those most relevant to the research questions are reported here. Both the 4-item Patient Health Questionnaire (PHQ-4) and 10-item Patient Health Questionnaire (PHQ-D) were chosen to capture a broad range of psychological distress. The PHQ-4 provided a quick screen for anxiety and depression, while the PHQ-D, including the PHQ stress module, offered deeper insight into specific psychosocial stressors, such as relationship difficulties, financial concerns, and work-related stress. This combination ensured efficient screening and a broad assessment of distress, anxiety, and depression in the general population (30). The following assessment instruments were selected to operationalize the constructs of the perception of AI and digital therapy, mental health, bonding, and affective engagement.

#### 2.4.1 Perception of AI and digital therapy

##### 2.4.1.1 Motives and expectations

Participants were asked about their “motives for using Clare” and their “expectations of using Clare,” and multiple answers could be given. Motives and expectations for AI-based mental health support were assessed using 11 predefined options each. The expectation list (e.g., fast and easy access, anonymity, and reduced emphasis on appearance) and motive list (e.g., problem-solving opportunities, gaining clarity, and obtaining information) were adapted from Eichenberg (31), who used them in an online self-help study. These lists were translated from German to English (see Supplementary Tables B.1 and B.2 for all items and abbreviations).

##### 2.4.1.2 Attitudes Toward Psychological Online Interventions (APOI)

The Attitudes toward Psychological Online Interventions Questionnaire (APOI) assesses the attitudes toward psychological online interventions in general (32). The questionnaire includes four subscales: skepticism and risk perception, confidence in effectiveness, technologization threat, and anonymity benefits. The anonymity subscale evaluates users' views on online interventions vs. traditional therapy. Participants respond to 16 statements using a dichotomous scale.

#### 2.4.2 Mental health outcomes

##### 2.4.2.1 PHQ-4

The PHQ-4 assesses depression and anxiety, with items from PHQ-2 and the Generalized Anxiety Disorder scale (GAD-2) rated on a 4-point scale (33). A score of 3 or higher on any subscale indicates a positive screening result, while the total PHQ-4 score (ranging from 0 to 12) classifies psychological distress as none (0–2), mild (3–5), moderate (6–8), or severe (9–12).

##### 2.4.2.2 UCLA Loneliness Scale (3-item version)

The 3-item UCLA Loneliness Scale is a shortened version of the original scale assessing loneliness and social isolation (34). Participants respond to 3 items using a 4-point scale (1 = never, 2 = rarely, 3 = sometimes, 4 = always). The responses are reverse-coded where appropriate, and the final score is calculated by averaging the ratings, with higher scores indicating greater loneliness.

##### 2.4.2.3 PHQ-D stress module (items 121 – 12j in the PHQ-D)

The PHQ stress module, a component of the PHQ-D, consists of 10 items assessing psychosocial stressors such as relationship difficulties, financial concerns, and work-related stress. Participants rate each item on a scale from 0 (not affected) to 2 (severely affected), yielding a total score between 0 and 20. The score reflects the severity of stress: 0–4 (minimal), 5–9 (mild), 10–14 (moderate), and 15–20 (severe) (35).

##### 2.4.2.4 3-item Social Phobia Inventory

The 3-item Social Phobia Inventory (Mini-SPIN) is a brief version of the Social Phobia Inventory assessing fear, avoidance, and physiological distress. Participants rate three items on a 5-point scale (0 = not at all, 4 = extremely), and a cutoff score of 6 or greater has been suggested for social anxiety (36).

##### 2.4.2.5 Satisfaction with Life Scale

The Satisfaction with Life Scale (SWLS) is a brief 5-item measure of global life satisfaction. Items are rated on a 7-point scale (1 = strongly disagree to 7 = strongly agree), yielding a total score between 5 and 35. A score of 20 suggests a moderate level of life satisfaction, with higher scores indicating greater satisfaction (37).

#### 2.4.3 Bonding and affective engagement

##### 2.4.3.1 Working Alliance Inventory-Short Revised

Participants self-reported affective engagement and bonding with the AI using the 12-item WAI-SR. This is a measure of subjective therapeutic alliance, including subscales for bond, goal, and task (38), based on Bordin's (39) working alliance theory. “Therapist” was replaced with “Clare®” to assess the user alliance with the relational agent (Supplementary Table C.1), following prior research approaches on CAI and therapeutic bonding (17, 40). Per the methods of Jasper et al. (41), bond scores of ≥3.45 were considered high, as seen in Darcy et al. (17).

### 2.5 Data analysis

Data were analyzed using IBM SPSS Statistics Version 28. Descriptive statistics summarized sociodemographic characteristics, clinical symptoms (UCLA, PHQ-D, PHQ-4, SWLS, and Mini-SPIN), and prior experience with digital mental health tools. Attitudes toward online therapy (APOI) were reported as percent agreement on a dichotomous scale (yes/no). Agreement with motives and expectations was assessed with yes/no answers and analyzed by ordering items based on the percentage of affirmative responses.

The t1b sample (*n* = 348) was descriptively analyzed for working alliance (WAI-SR) and its correlation with loneliness (UCLA). Bivariate Pearson correlation coefficients were calculated to assess the strength and direction of the relationships between working alliance (WAI-SR) and loneliness (UCLA), and with satisfaction with life (SWLS), anxiety and depression (PHQ-4), stress (PHQ-D), and social anxiety (Mini-SPIN). A two-sided independent *t*-test was conducted to compare WAI-SR scores between women (*n* = 176) and men (*n* = 168).

To examine the differences between completers and non-completers, a dropout analysis was conducted. Descriptive statistics (age and sex) were reported for both groups. Given the unequal sample sizes and violation of the homogeneity of variances (as indicated by Levene's tests), Welch's *t*-tests were used to compare mental health indicators (e.g., depression, anxiety, social anxiety, loneliness, and working alliance) between groups.

## 3 Results

### 3.1 Participant characteristics

Of the 604 people screened, 527 met the study criteria and were included in the baseline sample (t1). The mean age was 36.2 years (SD = 9.39; range = 18–64 years) as described in Table 3.

**Table 3:** Sociodemographic characteristics of the participants at baseline (t1; *n* = 527).

Baseline characteristic	*n*	%
Gender		
Female	277	52.56
Male	245	46.49
Diverse	4	0.76
I prefer not to say	1	0.19
Ethnicity		
White	397	75.33
African American	53	10.06
Asian	30	5.69
Hispanic	20	3.80
Mixed	20	3.80
Other	5	0.95
I choose not to say	2	0.38
Birthplace (country)		
United Kingdom	207	39.28
Germany	158	29.98
United States	139	26.38
Other	10	1.89
I choose not to say	13	2.47
Family status^a^		
Single	204	38.71
Cohabiting	49	9.30
Married	255	48.39
Separated/divorced	15	2.85
Widowed	14	2.66
Highest education		
Middle school	18	3.42
Apprenticeship	28	5.31
High school/A-level	295	55.97
University degree	165	31.31
Other	21	3.99
Employment status		
Employed	443	84.06
Unemployed	33	6.26
Retired	24	4.55
Homemaker	4	0.76
Other	18	3.42
I choose not to say	5	0.95
Profession		
Student	61	11.58
Employee	304	57.69
Civil servant	68	12.90
Self-employed	68	12.90
Unemployed	19	3.61
Other	7	1.33
Work setting		
Office	226	42.88
Home office	104	19.73
Both work settings	146	27.70
I choose not to say	51	9.68
Habitat^a^		
Shared Living	202	38.33
Alone	159	30.17
Campus dorm	68	12.90
With parents	61	11.57
Other	45	8.54
Experience with digital mental health treatment^b^		
Current use of other tools	8	1.52
Having interacted with Clare® before	7	1.33

*N* = 527. Participants were on average 36.20 years old (SD = 9.39, range = 18–64).

a Participants could choose more than one option.

b Reflects the number and percentage of participants answering “yes” to this question.

#### 3.1.1 Baseline clinical symptomatology

An overview of participants' scores for the clinical variables (UCLA, PHQ-D, PHQ-4, SWLS, Mini-SPIN) is presented in Table 4. The mean PHQ-4 score was 7.9 (SD = 2.14), with 37% showing a moderate elevation (score = 6–8) and 47.4% exhibiting a severe elevation (score = 9–12) in anxiety and depression symptoms. For 68.7% of participants, the PHQ-4 scores indicated anxiety disorders, while 59.2% showed scores suggesting depressive disorders (≥3 on PHQ-4 subscales). The mean PHQ-D score was 12.6 (SD = 3.31), with 54.1% having moderate psychosocial stress (score 6–8) and 31.9% experiencing severe stress (score 9–12). The mean UCLA score was 6.95 (SD = 1.46), with 85.9% classified as “lonely” (score ≥6). The mean SWLS score was 21.2 (SD = 7.10), with 20.3% being (extremely) dissatisfied (score ≥10) and 30.2% (extremely) satisfied (score ≥26) with their life. The mean Mini-SPIN score was 8.70 (SD = 2.38), with 84.4% at risk for generalized social anxiety disorder (score ≥6).

**Table 4:** Descriptive statistics and correlations for clinical variables (t1; *n* = 527).

Variable	*M*, SD	Range	Median	Mode
UCLA	6.95^a^, 1.46	3–9	7.00	8.00
PHQ-D	12.65^b^, 3.31	0–20	13.00	16.00
PHQ-4	7.90^c^, 2.41	0–12	8.00	9.00
SWLS	21.23^d^, 7.10	5–35	23.00	23.00
Mini-SPIN	8.70^e^, 2.38	0–12	9.00	9.0

UCLA, UCLA Loneliness Scale; PHQ-D, 10-item Patient Health Questionnaire; PHQ-4, 4-item Patient Health Questionnaire; SWLS, Satisfaction with Life Scale; Mini-SPIN, 3-item Social Phobia Inventory.

a Scores ≥6 are classified as “lonely” (UCLA).

b Scores reflect moderate 10–14 to severe stress 15–20 (PHQ-D).

c Scores reflect moderate psychological distress, anxiety, and depression (scores 6–8, PHQ-4).

d Scores ≥10 classy as (extremely) dissatisfied, score ≥ 26 as (extremely) satisfied with one’s life.

e Scores ≥6 are classified as at risk of a generalized social anxiety disorder.

### 3.2 Engagement patterns

We explored participant engagement by examining call frequency and duration across both mid-treatment (*n* = 53) and post-treatment (*n* = 21) samples. Engagement was tracked over a 4-week period in the mid-treatment phase and an 8-week period in the post-treatment phase. For a detailed overview, refer to Supplementary Tables E.1–E.4.

#### 3.2.1 Call engagement over time

In the mid-treatment sample (*n* = 53), both the frequency and duration of calls declined over the 4 weeks. The average number of calls in week 1 was 1.77 (SD = 1.52). Call frequency dropped to 0.68 (SD = 0.96) in week 2 and continued to decrease in week 3 to 0.49 (SD = .64) and in week 4 to 0.40 (SD = .57), indicating a reduction in participant engagement over time. A similar decline was observed in call duration. In week 1, the average call length was 3.35 min (SD = 4.37), decreasing to 2.01 (SD = 3.23) in week 2, 1.25 (SD = 2.55) in week 3, and 1.45 (SD = 2.95) in week 4.

#### 3.2.2 Call engagement in the post-treatment sample

Participants in the post-treatment sample (*n* = 21) showed sustained interaction with Clare® across the full 8-week period. In week 1, the average number of calls was 2.10 (SD = 1.48). Although call frequency declined over time, the participants received approximately one call per week through week 5. A more noticeable drop occurred in weeks 6 and 7, with call frequency at 0.52 (SD = 0.75), followed by a slight recovery in week 8 to 0.57 (SD = .51). Call duration followed a similar trend. In week 1, the average call lasted 3.96 min (SD = 5.10). The duration remained relatively high through week 4 (range: 2.41–3.25 min), declined in weeks 5–7, and rebounded slightly in week 8 to 2.90 min (SD = 4.34).

### 3.3 Dropout analysis

Comparisons between completers (*n* = 21) and non-completers (*n* = 348) showed that non-completers reported significantly higher mental health distress, with most completers being female. Both groups initially formed a strong working alliance with the bot, but non-completers did not maintain engagement. Similar patterns were found when comparing individuals who completed t2 (*n* = 53) to non-completers (*n* = 316). A detailed overview of the dropout analysis is provided in Supplementary Material.

### 3.4 Previous experience and attitudes toward psychological online interventions

**Figure 2:**
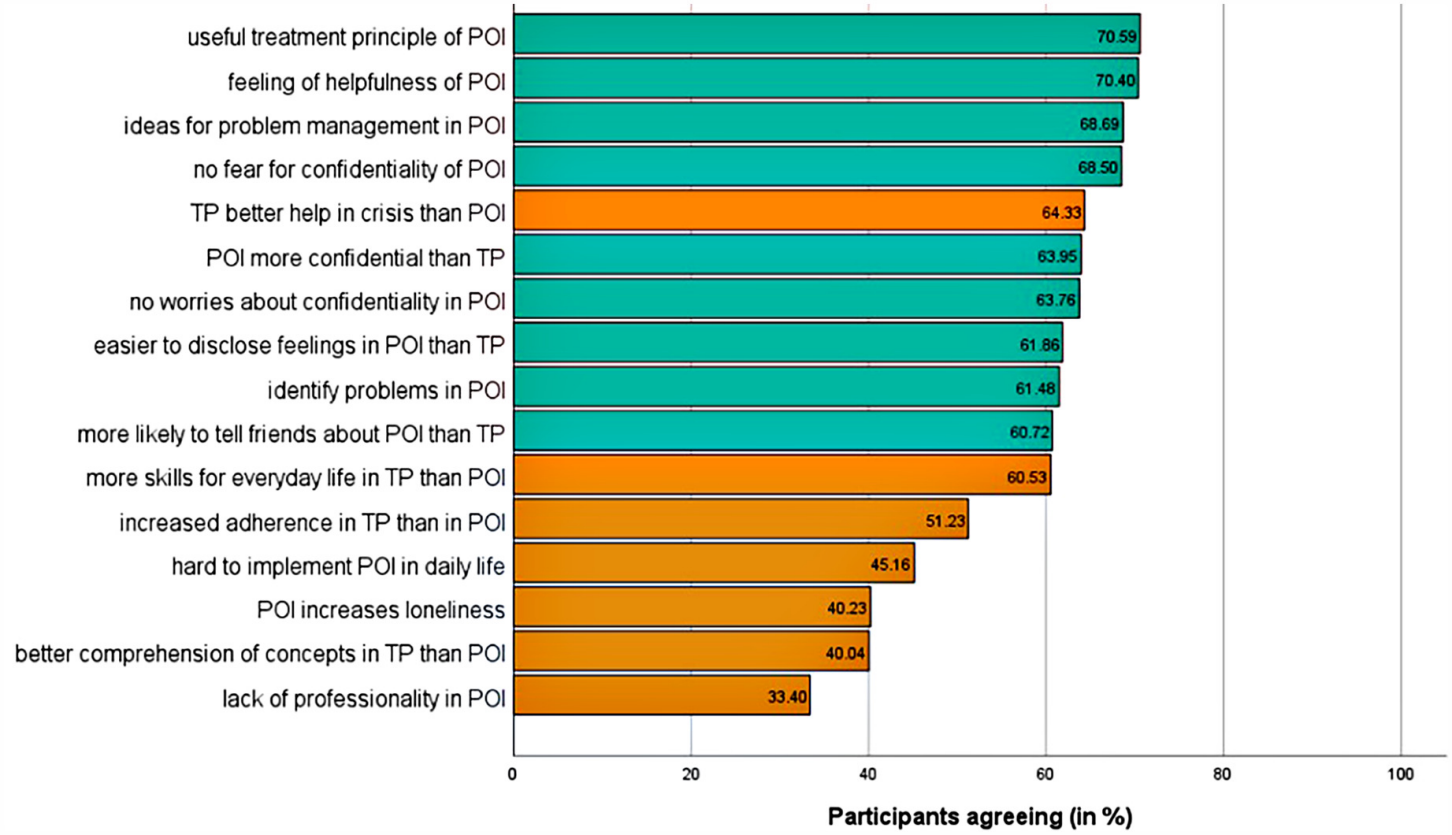
Attitudes toward Psychological Online Interventions Questionnaire (APOI; yes-votes in order of decreasing agreement; t1; *n* = 527). Items are abbreviated for illustrative reasons. Abbreviations are defined in Supplementary Table A.1. Answers are on a dichotomous scale (yes/no). The selection of multiple motives was possible. Green indicates positive and orange negative attitudes toward psychological online interventions. POI, psychological online interventions; TP, traditional psychotherapy.

Of the 527 participants, only 8 (1.52%) had used other digital mental health tools, and 7 (1.33%) had previously interacted with Clare®. Positive attitudes toward online interventions were generally more prevalent than negative ones, except for crises, where 64.33% (*n* = 339) favored traditional psychotherapy (Figure 2).

### 3.5 Motives and expectations of AI therapy

**Figure 3:**
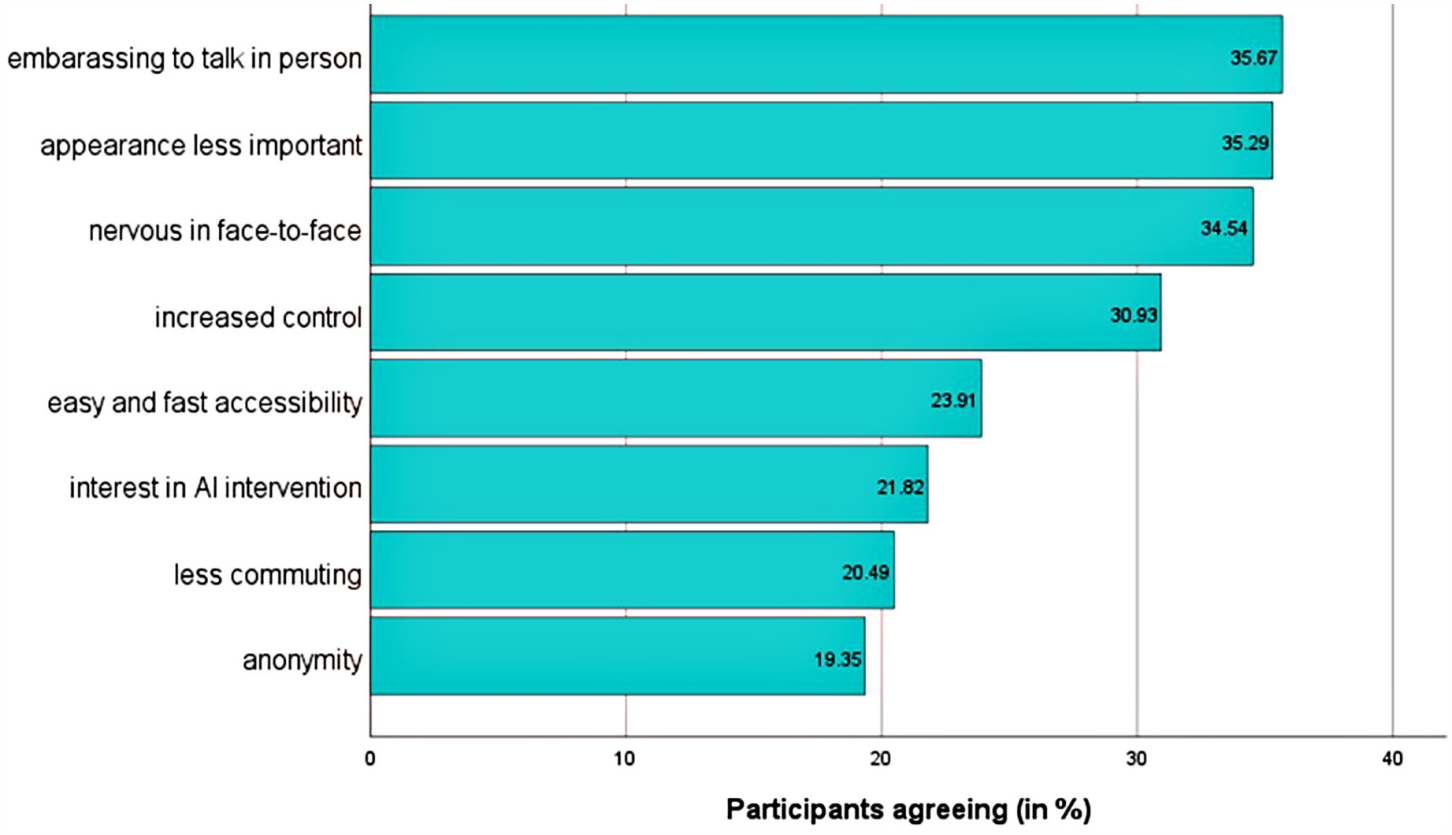
Motives for seeking AI advice (yes-votes in order of decreasing agreement; t1; *n* = 527). Items are abbreviated for illustrative reasons. Abbreviations are defined in Supplementary Table B.1. Answers are on a dichotomous scale (yes/no). The selection of multiple motives was possible.

**Figure 4:**
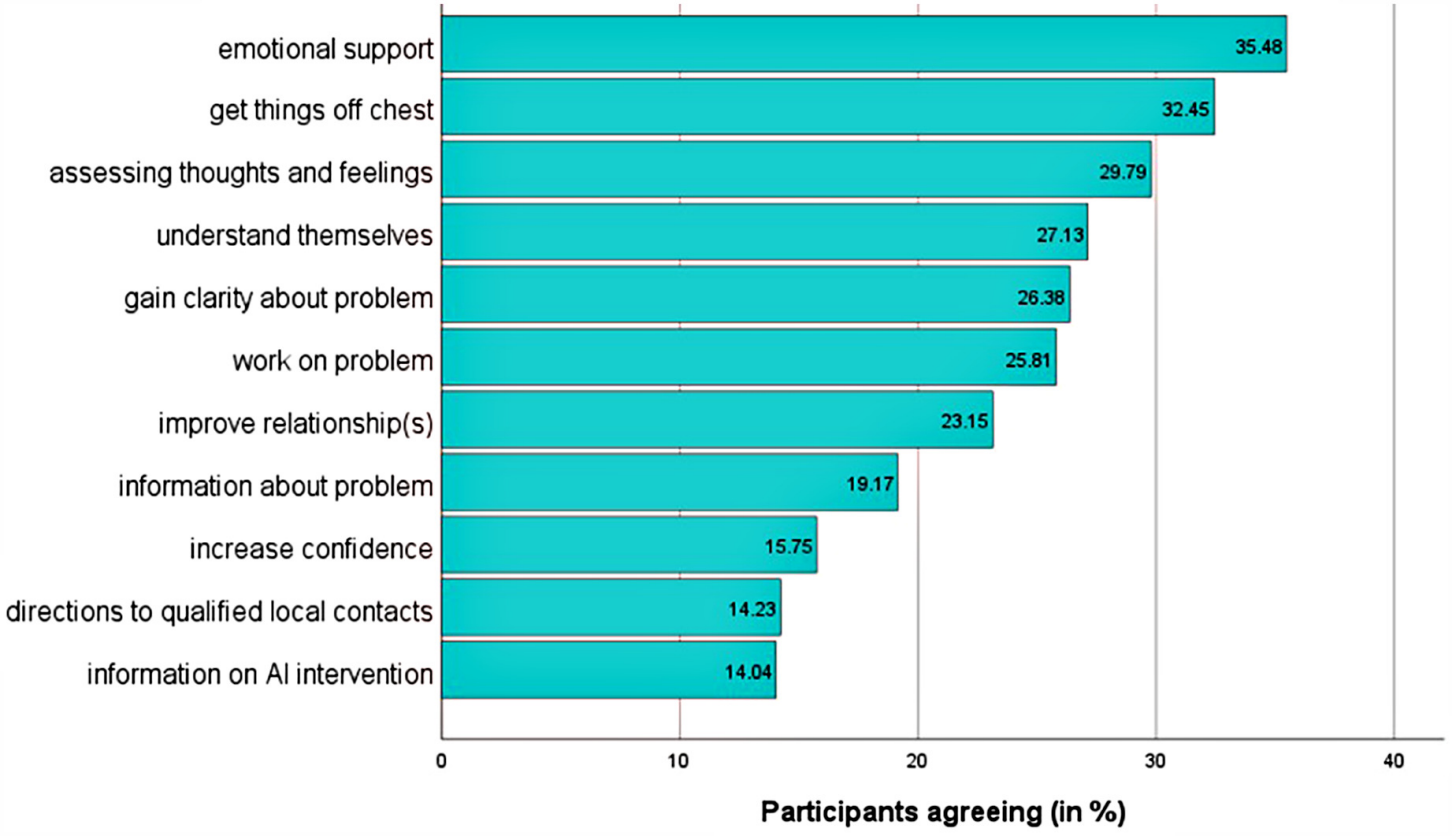
Expectations of AI advice (yes-votes in order of decreasing agreement; t1; *n* = 527). Items are abbreviated for illustrative reasons. Abbreviations are defined in Supplementary Table B.1. Answers are on a dichotomous scale (yes/no). The selection of multiple motives was possible.

The primary motives were avoiding embarrassment when discussing problems with humans (35.7%, *n* = 188) and receiving advice regardless of appearance (35.29%, *n* = 186). Lesser motives included reduced commuting (20.5%, *n* = 108) and anonymity (19.6%, *n* = 102) (Figure 3). The main expectations were emotional support (35.5%, *n* = 187) and expressing feelings (32.5%, *n* = 171). Fewer participants expected referrals to local contacts (14.3%, *n* = 75) or information on AI self-help (14%, *n* = 74) (Figure 4).

After 3–5 days (t1b), users reported their working alliance with Clare® (WAI-SR; *M* = 3.76; SD = 0.72; see Table 5). All the subscales of the working alliance—total, goal, task, and bonding—showed positive and significant correlations with loneliness (Table 6). Male participants (*n* = 168) reported higher mean scores (*M* = 3.88) than female participants (*n* = 176, *M* = 3.65) for overall working alliance. The difference between the groups was small to moderate [*t* (348) = −3.17, *p* = 0.002, *d* = −0.34], as was the difference in the goal [*t* (348) = −2.40, *p* = .017, *d* = −0.26], task [*t* (348) = −4.09, *p* < 0.001, *d* = −0.44], and bonding subscales [*t* (342) = −2.14, *p* = 0.033, *d* = −0.23].

**Table 5:** Bonding experience after 3–5 days of using Clare^®^.

Working alliance inventory- short revised – subscale	Mean (SD)	Range
Total score	3.76 (0.72)^a^*	1–5
Goal subscale	3.73 (0.83)^a^	1–5
Task subscale	3.74 (0.78)^a^	1–5
Bonding subscale	3.82 (0.77)^a^	1–5

*N* = 348; t1b sample assessed 3–5 days after using Clare^®^.

a Per the method of Jasper et al. (41), bond scores of ≥3.45 were considered high.

**Table 6:** Association between loneliness at baseline and bonding experience after 3–5 days of using Clare^®^.

Subscale of working alliance inventory- short revised subscale correlated with UCLA loneliness scale	*r*	*p*
Total	0.25	<.001
Goal	0.21	<.001
Task	0.25	<.001
Bonding	0.21	<.001

*N* = 348; t1b sample assessed 3–5 days after using Clare^®^.

Furthermore, the total working alliance scores (WAI-SR) showed statistically significant low positive correlations with psychosocial stress (PHQ-D; *r* = .337, *p* < .001), anxiety and depression (PHQ-4; *r* = .368, *p* < .001), and social anxiety (Mini-SPIN; *r* = .336, *p* < .001). A negligible, but still significant, positive correlation was also observed with life satisfaction (SWLS; *r* = .097, *p* = .041). Correlation strengths are interpreted according to the classification in a previous study (42).

## 4 Discussion

The presented study explored the characteristics of individuals interested in conversational AI for self-help, and their psychological wellbeing, motives, and expectations of AI therapy, and the attitudes toward online therapy in general among users of a conversational AI for mental health support (Clare®), and their working alliance and bonding with Clare®.

### 4.1 Demographic profile of users of CAI

#### 4.1.1 Age and gender

In this study, 53% of participants identified as female, closely aligning with findings by Darcy et al. (17) with 57.5% female participation of self-referred users of the CBT-based conversational agent Woebot® within an age range of 18–78 years. Studies on AI interventions with self-referred samples are scarce, often focusing on college or clinical populations. For example, Fitzpatrick et al. (16) reported an average age of 22.2 years (67% female) among college students, while another study (43) reported a mean age of 22.9 years (70% female) in a clinical sample using the AI Tess®. This reflects broader trends where women are more likely to seek mental health services. Unlike previous CAI studies that focused on young female adults (12, 16, 44), our sample shows a balanced gender distribution and spans a broad age range. This may suggest that chatbots such as Clare® may effectively address mental health issues across genders, potentially reducing stigma and enhancing participation. While both men and women are attracted to AI for self-help, gender-specific approaches could further improve the effectiveness of digital mental health interventions. Additionally, our findings challenge the notion that AI is primarily a solution for Generation Z (45), indicating that AI tools can enhance mental health support for many generations.

#### 4.1.2 Psychological wellbeing of users of Clare® at baseline

The sample exhibited high anxiety and depression levels, comparable to rates in the general populations of the UK, Germany, and the US (46–48). Despite this, no acute crises were detected during onboarding. Moderate psychosocial stress was reported by half of the participants, and severe stress by 32%. Loneliness was significant, affecting 86% of the participants, raising public health concerns (49, 50). Loneliness is recognized as detrimental to health, and while CAIs, including chatbots, have the potential for social support, their impact on loneliness remains under-researched (9), but could be a central therapeutic target of CAIs.

#### 4.1.3 Drop-out and engagement patterns

This study found that individuals with higher levels of distress were less likely to stay engaged with the AI-based support, aligning with the bot's intended scope for moderate distress. While initial working alliances were strong, sustained engagement proved challenging, suggesting the importance of maintaining this connection for continued participation.

Differences in engagement patterns suggest that sustained contact plays a critical role in participant retention. While the mid-treatment sample showed a sharp drop in call frequency and duration after week 1, the post-treatment sample maintained more consistent engagement across all 8 weeks. These findings highlight the importance of early and ongoing interaction in supporting adherence and reducing dropout.

#### 4.1.4 Attitudes toward digital mental health support

We observed high acceptance of psychological online therapy, with 71% finding it effective, useful, and helpful. However, only 3% reported using other digital mental health support. Previous research indicates that favorable attitudes do not always lead to high engagement (51, 52), and initial interest often fades due to the “novelty effect” (53). To be considered effective, interventions should aim for positive long-term effects (54). User engagement with Clare® may be partly influenced by a novelty effect. While we examined whether the working alliance was sustained over time, we did not directly assess this effect.

Future studies should aim to distinguish novelty-driven from sustained engagement, for example, by comparing early and later outcomes, including a familiar control group, or using a mixed methods approach to gain a deeper behavioral understanding—separating novelty-period responses from sustained motivation or engagement (53).

Furthermore, positive attitudes are linked to better implementation and efficacy (55, 56). Therefore, managing expectations, ensuring transparency, and educating users on CAI's limitations and possibilities are vital for sustaining its positive impact. These measures may prevent negative experiences and attitudes that may undermine the positive effects of such interventions. Interestingly, 69% of participants had no confidentiality concerns with online therapy, and 64% found online platforms more secure than traditional methods. While previous guidelines emphasize confidentiality in e-therapy (57), this was less of a concern for Clare® users. Additionally, 62% found it easier to disclose feelings online and were more likely to discuss online therapy with friends than traditional therapy. AI-driven self-help tools may offer a valuable alternative for individuals who find conventional counseling or mHealth interventions inaccessible.

Approximately 40% of the participants worried that online interventions may worsen their loneliness, a concern relevant as many users were already experiencing significant loneliness. This concern reflects broader debates on AI and social isolation; while some see human-AI interactions as a remedy, others warn of increased societal withdrawal (58). Critics suggest that although CAI may temporarily ease isolation, over-reliance could deepen loneliness and alienate individuals from human contact (59), raising concerns about “AI delusion,” and the need for safe AI responses (26). Additionally, 33% questioned the professionalism of digital mental health tools and 64% preferred traditional therapy over online options in crisis, highlighting the need for further research and careful management of expectations of CAI for mental health.

### 4.2 Motives and expectations of AI self-help

The primary motivations for seeking AI-based mental health support included avoiding face-to-face contact, managing nervousness and embarrassment, and maintaining control. Participants expected that AI would offer emotional support, facilitate self-expression, aid in self-assessment, and improve relationships. Other benefits included increased confidence and knowledge of local support options.

First encounters with therapists can trigger insecurities and feelings of shame (60), a universal emotion linked to perceived inadequacy (61). Shame has been a barrier in therapeutic contexts, particularly for trauma-related cases (62) or male patients (63). Research indicates that AI chatbots can reduce feelings of shame, nervousness, and distress, promoting the realization that seeking help is not shameful (64). Moreover, a recent study showed that there is no difference in self-reported intimacy of self-disclosure between human and chatbot conditions. While chatbots were associated with less fear of judgment, humans were perceived as more trustworthy (65).

This may imply that conversational AI systems should prioritize emotionally safe, non-judgmental environments to facilitate self-disclosure and reduce help-seeking barriers. Despite comparable levels of disclosure to chatbots and humans, lower trust in chatbots underscores the need for transparent design, consistent behavior, and empathetic responses. Incorporating human-in-the-loop mechanisms and human-centered design approaches for bots (23, 66) can enhance oversight and trust. As the exact purpose of bots and the nature of the relationship a user has with them are not always obvious and understood (13), user education and digital literacy are essential for managing expectations and supporting informed use. CAIs may serve as effective adjuncts to traditional care, particularly in early engagement.

Contrary to earlier findings, anonymity and personalized information were less crucial for Clare® users. While anonymity often enhances self-disclosure in digital settings (67), recent studies suggest that perceived emotionality in chatbots can reduce self-disclosure (68). This highlights the potential benefits of AI's limited emotional engagement and artificiality.

### 4.3 Working alliance with Clare®

Our preliminary findings suggest that users develop a strong bond with Clare® within 3–5 days, indicating an initial positive working alliance. This effect appears particularly pronounced in individuals with high loneliness scores. Moreover, we found that a stronger working alliance was moderately associated with a higher symptom burden. Given the well-documented link between the therapeutic alliance and positive psychotherapy outcomes (69), further research is needed to examine the stability and clinical relevance of this bond over time. This level of bonding is consistent with other conversational agents such as Wysa® and Woebot® (17, 70). Beatty et al. (70) reported a mean WAI-SR score of 3.64 (SD 0.81) among users (*n* = 1,205), and Darcy et al. (17) observed similar scores among Woebot® users (*n* = 36,070), with a mean bond subscale score of 3.8 (SD 1.0), where bond scores of ≥3.45 are considered high (41). These working alliance scores are comparable to in-person outpatient psychotherapy (71) and group CBT (41).

Notably, our study found higher bonding scores among male participants (*M* = 3.9) compared to women (*M* = 3.7), contrasting with Darcy et al. (17), where higher scores were reported among women. The finding of higher bonding scores among male participants may reflect that men often report greater difficulty talking to mental health professionals, such as psychologists (72). Men may feel more comfortable engaging with AI-driven therapy. Shame linked to a psychotherapeutic dialogue (73) and less fear of perceived judgement in AI interactions (65) may reduce barriers to emotional expression, fostering stronger bonds. Tailoring AI interventions to address gender-specific needs could improve engagement and accessibility, especially for men. Higher loneliness in users correlated with stronger bonding with Clare®, linking AI bonding to clinical variables. This highlights AI's potential to meet emotional needs and emphasizes the importance of considering dependency and unhealthy attachments in users with mental health issues (74).

The early formation of a bond with the AI agent appears promising, but raises important questions about its long-term stability. Notably, early therapeutic alliance ratings have shown limited predictive value, highlighting the need to conceptualize the alliance as a dynamic process that unfolds over the course of treatment. In internet-based cognitive behavioral therapy (ICBT) for depression and anxiety, evidence on the relationship between the alliance and clinical outcomes remains mixed; while some studies report no significant association (75, 76), others found correlations at various stages, including early (77) and mid-treatment (78, 79). These inconsistencies suggest that the function and formation of the therapeutic alliance may differ in digital compared to face-to-face settings. Future research should investigate whether the alliance formed with digital agents is sustained over time and whether it contributes meaningfully to clinical improvement.

In this study, the WAI-SR was adapted by replacing “therapist” with the AI agent “Clare®,” allowing for the assessment of alliance-like perceptions in a digital context. However, this raises concerns about the construct validity of relational constructs, such as “bond,” when interacting with non-human agents. While alliance scores may reflect perceptions of responsiveness or trustworthiness, their comparability to traditional human-delivered therapy is limited. Future research should explore the validity of these constructs in digital interactions.

### 4.4 General implications of our results for CAI

While Clare® offers promising potential in mental health applications, it is essential to acknowledge the ongoing ethical and safety concerns surrounding AI in therapeutic contexts. Issues such as patient autonomy, over-reliance, and trust remain significant challenges (13, 24, 80). The use of proactive conversational agents capable of topic shifting and generating novel treatment plans (19) presents additional complexities, including ensuring conversational safety, preventing inappropriate responses, and maintaining alignment with therapeutic objectives. Clare® was developed through interdisciplinary collaboration involving data engineers, developers, researchers, psychologists, and conversation designers to address these concerns and key ethical principles (see Section 2.1.2).

The use of AI in caregiving, especially in clinical and therapeutic settings, raises critical ethical and safety concerns about the effectiveness of psychological support and human-AI relationships. These concerns are particularly pronounced for vulnerable groups, such as low-income or minority populations, where emotional dependency on AI could pose substantial risks (81, 82). This highlights the need for stringent ethical standards (14, 83, 84). CAIs, especially those based on LLMs, face significant safety and ethical challenges, including preserving patient autonomy, reducing manipulation risks, and ensuring proper user-technology relationships and privacy (13, 24).

Users' expectations of AI mental health support are not fully understood, raising concerns about AI capabilities and the potential for misunderstanding or deception. Ensuring safety, reliability, risk management, and expectation clarity is crucial (14). Proactive CAI systems may be seen as intrusive, affecting user comfort (66). Ethical issues, such as biased training data (85) and harmful advice (86), can harm user outcomes and erode public trust (23). Clear guidelines and risk assessments are needed to address these concerns and ensure responsible AI use, as demonstrated by models such as Clare®, which incorporates safety measures and human support connections.

### 4.5 Limitations and future studies

While this baseline data provides valuable insights, several limitations should be considered: the sample was predominantly from Western countries (the UK, Germany, and the US), which limits the generalizability of the findings to other cultural contexts. Attitudes toward mental health, trust in digital technologies, and communication norms vary across cultures (87), influencing user engagement and perceptions of CAI. Misunderstandings about a chatbot's purpose or differing care expectations can also affect engagement and perceived usefulness (13). Socio-cultural and political contexts further shape expectations and concerns about AI. Embedding cultural values is therefore essential (88). Future research should aim to recruit more diverse samples by partnering with international research institutions, translating interventions into multiple languages, and adapting content to be culturally sensitive. These steps help ensure CAIs are effective and acceptable across a wide range of cultural contexts.

The recruitment method may have led to an inherent bias, particularly in terms of digital access, language proficiency, and platform-specific reach. Social media platforms differ in user demographics and usage patterns; to mitigate this bias and foster sample diversity, we deliberately employed a range of platforms to engage participants from varied age groups, gender identities, and educational backgrounds.

Given that only 1.52% of participants had used other digital mental health tools, and only 1.33% had interacted with Clare® previously, the novelty of the platform may have influenced initial perceptions and alliance ratings. Users unfamiliar with such tools may have rated their alliance more positively due to a lack of comparison with other platforms. Future studies should examine how prior experience with digital mental health tools may shape user expectations and alliance development, the sustainability of the working alliance over time, and whether familiarity leads to different patterns of engagement and alliance formation.

Adapting the WAI-SR for AI interactions may affect the interpretability and construct validity of the alliance subscales, particularly the bond dimension. The results should, therefore, be interpreted with caution, and future work should explore whether new alliance measures tailored to digital agents are warranted.

This baseline paper relies on self-reported data, which can be subject to biases such as social desirability and recall bias. Furthermore, self-reported data is limited, as we did not ask for a diagnosis or prior or current treatment. Another limitation of this study is the use of surveys administered every 4 weeks instead of Ecological Momentary Assessment (EMA), which could better minimize recall bias, enhance ecological validity, and capture real-time behaviors in natural environments in future research (89, 90).

Moreover, questions remain about the sustainability of the therapeutic alliance of the CAI with its users over time, which needs further investigation. Given the exploratory nature of this study, participant attrition was expected. Future research should investigate factors contributing to attrition, including demographic and psychological variables, and explore strategies to improve engagement, especially for those with higher distress. A limitation of the study is that expectations towards Clare® were only assessed at the beginning of participation (t1). Therefore, it is not possible to determine whether expectations changed over time or if they were influenced by the actual usage experience. While a comparison of expectations between the completers and dropouts could be made, it remains unclear to what extent these expectations influenced actual usage behavior or dropout.

The scope of this baseline paper is limited to reporting baseline data and initial working alliance, assessed 3–5 days after onboarding, a timeframe consistent with prior studies (16, 70, 79). Interaction data (e.g., call duration and frequency per week) is also included. Follow-up measurements will be presented in a subsequent publication. This will include the mobile Agnew Relationship Measure (mARM), a measure of the therapeutic relationship, which was administered only after the participants had interacted with Clare® (at weeks 4 and 8, i.e., t2 and t3). The mARM assesses experiences with digital mental health interventions. Based on a review of existing research, no studies were found that employed this measure after a short period of interaction. While both the mARM and WAI-SR assess the therapeutic alliance, the WAI-SR has been used in digital mental health studies following brief interactions (3–5 days), supporting its cross-sectional applicability. Given the novelty of this field, a review of the item content led to the conclusion that the mARM requires more prolonged interaction to yield valid measurements and is most meaningful after at least 4 weeks of use, which is a key methodological consideration for future research.

A future paper will explore the users’ interaction data alongside changes in psychological distress and therapeutic alliance over 4- and 8-week periods. Given the small sample size, analyses will be primarily descriptive, supplemented by *t*-tests to explore potential group differences. Variables of interest will include alliance scores and changes in psychological distress. In addition, subgroup analyses will examine usage frequency and its association with these outcomes, offering preliminary insights into potential patterns of engagement and effect.

## 5 Conclusions

Users with mental health challenges are attracted to and bond with CAI for self-help, seeking emotional support without concerns about shame or physical appearance. Future research should examine change mechanisms, such as the working alliance, and their impact on the overall wellbeing of users. Addressing ethical considerations, relationship dynamics, and risk management is crucial for effective CAIs. Understanding user expectations and user behavior of CAI will enhance education on AI capabilities and limitations.

## Data Availability

The raw data supporting the conclusions of this article will be made available by the authors, without undue reservation.
