# Supplementary material for: Exploring user characteristics, motives, and expectations and the therapeutic alliance in the mental health conversational AI Clare®: a baseline study
Source: Front Digit Health. 2025 Jun 13;7:1576135. doi: 10.3389/fdgth.2025.1576135 (PMC12203671; doi:10.3389/fdgth.2025.1576135)
Supplement: Supplementary file 1 [file Supplementaryfile1.docx]

**Appendices to Exploring User Characteristics, Motives and Expectations and Therapeutic Alliance in the Mental Health Conversational AI Clare®: A Baseline Study**

# Appendix A.

Abbreviations used in Figure 5 for items of the Attitudes towards psychological online therapy (APOI) questionnaire.

**Table A.1**

*Abbreviations for Attitudes towards psychological online therapy (APOI) questionnaire.*

| Item | Abbreviation |
| --- | --- |
| I would be more likely to tell my friends / acquaintances about using an online psychological intervention than about psychotherapy. | more likely to tell friends about POI than TP |
| An online psychological intervention is more confidential and discreet than psychotherapy with a therapist. | POI more confidential than TP |
| With a therapist, I am more likely to learn skills that help me cope better with everyday life than with an online psychological intervention. | more skills for everyday life in TP than POI |
| I think the treatment principle of online psychological interventions is basically useful. | useful treatment principle of POI |
| I feel that an online psychological intervention could help me. | feeling of helpfulness of POI |
| In my opinion, an online psychological intervention reinforces isolation and loneliness. | POI increases loneliness |
| It is difficult to actually implement the suggestions of an online psychological intervention in everyday life. | hard to implement POI in daily life |
| In psychotherapy with a therapist, I am more likely to “stay on the ball” than in an online psychological intervention. | increased adherence in TP than in POI |
| In an online psychological intervention, I do not receive professional support. | lack of professionality in POI |
| In an online psychological intervention, I cannot comprehend the theoretical therapy concepts as well as in psychotherapy. | better comprehension of concepts in TP than POI |
| An online psychological intervention can help me identify what problems I need to address and solve. | identify problems in POI |
| An online psychological intervention can give me important ideas on how to better manage my problems. | ideas for problem management in POI |
| In crisis situations, a therapist can help me better than an online psychological intervention. | TP better help in crisis than POI |
| With an online psychological intervention, I don’t have to worry about anyone finding out about my psychological problems. | no worries about confidentiality in POI |
| I find it easier to disclose my feelings with an online psychological intervention than with a therapist. | easier to disclose feelings in POI than TP |
| With an online psychological intervention, I don’t need to be afraid of anyone finding out about my psychological problems. | no fear for confidentiality of POI |

*Note.* POI = psychological online interventions; TP = traditional psychotherapy.

# Appendix B.

Abbreviations used in Figure 6 and 7 for Motives and Expectations of AI therapy.

**Table B.1**

*Abbreviations for Motives for seeking AI advice.*

| Item | Abbreviation |
| --- | --- |
| easy and fast accessibility | easy and fast accessibility |
| anonymity | anonymity |
| receiving advice regardless of personal appearance | appearance less important |
| better control over how much and what the advisor learns | increased control |
| embarrassment to talk about this problem with someone in person. | embarrassing to talk in person |
| too nervous to talk openly in a face-to-face counseling session | nervous in face-to-face |
| long commute to see a counselor in person | less commuting |
| would like to know how an AI intervention works | interest in AI intervention |

**Table B.2**

*Abbreviations for Expectations of AI advice.*

| Item | Abbreviation |
| --- | --- |
| opportunity to work on a problem that is really bothering me | work on problem |
| opportunity to gain clarity about my situations | gain clarity about problem |
| concrete information about my problem | information about problem |
| a realistic assessment of my thoughts and feelings | assessing thoughts and feelings |
| understanding me and my problems | understand themselves |
| suggestion for improving my relationship(s) | improve relationship(s) |
| an emotional support / relief | emotional support |
| a way to get things off my chest | get things off chest |
| directions to qualified local contacts | directions to qualified locals |
| more confidence to do things differently than before | increase confidence |
| information about what happens in AI self help | information on AI intervention |

# Appendix C.

Items of the Working Alliance Inventory-Short Revised (WAI-SR) questionnaire, in which the word “therapist” was changed to “Clare®” in adaptation for this study.

**Table C.1**

*Items of the Working Alliance Inventory-Short Revised (WAI-SR) questionnaire adapted to the study*

| Item number (Subscale) | WAI-SR Items |
| --- | --- |
| 1 (Task) | As a result of these sessions I am clearer as to how I might be able to change. |
| 2 (Task) | What I am doing with Clare® gives me new ways of looking at my problem. |
| 3 (Bond) | I believe Clare® likes me. |
| 4 (Goal) | Clare® and I collaborate on setting goals for this program. |
| 5 (Bond) | Clare® and I respect each other. |
| 6 (Goal) | Clare® and I are working towards mutually agreed upon goals. |
| 7 (Bond) | I feel that Clare® appreciates me. |
| 8 (Goal) | Clare® and I agree on what is important for me to work on. |
| 9 (Bond) | I feel Clare® cares about me even when I do things that it does not approve of. |
| 10 (Task) | I feel that the things I do with Clare® will help me to accomplish the changes that I want. |
| 11 (Goal) | Clare® and I have established a good understanding of the kind of changes that would be good for me. |
| 12 (Task) | I believe the way we are working with my problem is correct. |

**Appendix D.**

Dropout Analysis

Descriptive Statistics and Statistical Tests:

**1. Comparison of Completers and Non-Completers**

Completers: Participants who completed all surveys (T1, T1b, T2, T3) and interacted

with Clare® at least once between measurements.

- **Sample size: n = 21**
- Gender: Most completers were female (71.4%), with 5 males (23.8%) and 1 participant who did not specify gender.
- Age: The majority were aged 26–35 years (n = 8) and 36–55 years (n = 9).

Non-Completers: Participants who interacted with Clare®, completed T1 and T1b, but did not complete the T2 survey.

- **Sample size: n = 348**
- Gender: Equal distribution of male (50%) and female (50%), with 3 diverse.
- Age: Majority were 26–35 years (n = 130) and 36–55 years (n = 115), with 71 non-completers aged 18–25.

**Levene’s Test for Homogeneity of Variance:** Levene's test was conducted for each

measure to assess the assumption of equal variances. All measures indicated significant

violations of this assumption, thus, Welch’s t-test was employed for all comparisons.

**Welch’s t-test Results:**

**Depression and Anxiety (PHQ-4)**: Non-completers (M = 8.04, SD = 2.26) scored significantly higher than completers (M = 5.04, SD = 2.95), t(21.43) = –4.56, p < .001, d = 2.3 (very large effect size).

**Psychological Distress (PHQ-D)**: Non-completers (M = 12.70, SD = 2.9) scored significantly higher than completers (M = 8.80, SD = 2.26), t(21.89) = –5.23, p < .001, d = 2.9 (very large effect size).

**Social Anxiety (Mini-SPIN)**: Non-completers (M = 8.78) reported significantly higher social anxiety compared to completers (M = 7), t(21.49) = –3.28, p < .001, d = 2.3 (large effect size).

**Loneliness (UCLA)**: Non-completers (M = 6.97, SD = 1.44) reported significantly higher loneliness compared to completers (M = 5.80, SD = 2.20), t(21.05) = –3.45, p < .001, d = 1.5 (large effect size).

**Working Alliance (WAI-SR)**: Non-completers (M = 3.81, SD = 0.66) had a significantly higher working alliance compared to completers (M = 2.91, SD = 0.88), t(21.05) = –4.61, p < .001, d = 0.68 (medium effect size).

**2. Comparison of Completers vs. Dropouts at T2**

**Completers**: Participants who completed the T2 survey and interacted with Clare® at least once.

**Sample size: n = 53**

Gender: 39 females (73.6%), 11 males (20.8%), 2 diverse, and 1 unspecified.

Age: Majority were aged 26–35 years (n = 22) and 36–55 years (n = 19), with 12 completers aged 18–25.

**Non-Completers (Dropouts)**: Participants who dropped out at T2 after initial interactions with Clare®.

**Sample size: n = 316**

Gender: 71.8% female (n = 227), 26.9% male (n = 85), 1.3% unspecified.

Age: Majority were aged 26–35 years (n = 129) and 36–55 years (n = 112), with 75 non-completers aged 18–25.

**Welch’s t-test Results:**

**Depression and Anxiety (PHQ-4)**: Non-completers (M = 8.14, SD = 2.05) scored significantly higher than completers (M = 6.23, SD = 3.40), t(58.33) = –3.88, p < .001, d = 2.31 (very large effect size).

**Psychological Distress (PHQ-D)**: Non-completers (M = 12.81, SD = 2.83) scored significantly higher than completers (M = 10.47, SD = 3.90), t(61) = –4.18, p < .001, d = 3.0 (very large effect size).

**Social Anxiety (Mini-SPIN)**: Non-completers (M = 8.87, SD = 2.17) scored significantly higher than completers (M = 7.73, SD = 3.29), t(59) = –2.37, p < .001, d = 2.3 (large effect size).

**Working Alliance (WAI-SR)**: Non-completers (M = 3.88, SD = 0.60) had a significantly higher working alliance compared to completers (M = 3.19, SD = 0.90), t(61) = –5.15, p < .001, d = 0.67 (medium effect size).

**Outliers in Working Alliance:**

Several outliers were identified in the working alliance data, indicating that some

participants had unusually high or low levels of working alliance with the bot. These may

represent participants with unique characteristics or responses differing significantly from

the majority.

Both completers and non-completers initially formed a working alliance with the bot

(≥3.45, as suggested by Darcy et al., 2021). However, initial bonding did not appear to

guarantee ongoing engagement, suggesting the need for further investigation into the

sustainability of the working alliance.

**Table D.1**

*Statistical Results and Descriptive Data*

| Variable | Group | Mean (SD) | Welch’s t | df | p-value | Cohen’s d |
| --- | --- | --- | --- | --- | --- | --- |
| PHQ-4 | Completers (n=21) | 5.04 (2.95) | –4.56 | 21.43 | <.001 | 2.3 |
|  | Non-Completers (n=348) | 8.04 (2.26) |  |  |  |  |
| PHQ-D | Completers (n=21) | 8.80 (2.26) | –5.23 | 21.89 | <.001 | 2.9 |
|  | Non-Completers (n=348) | 12.70 (2.9) |  |  |  |  |
| Mini-SPIN | Completers (n=21) | 7.00 (NA) | –3.28 | 21.49 | <.001 | 2.3 |
|  | Non-Completers (n=348) | 8.78 (2.17) |  |  |  |  |
| UCLA Loneliness | Completers (n=21) | 5.80 (2.20) | –3.45 | 21.05 | <.001 | 1.5 |
|  | Non-Completers (n=348) | 6.97 (1.44) |  |  |  |  |
| WAI-SR | Completers (n=21) | 2.91 (0.88) | –4.61 | 21.05 | <.001 | 0.68 |
|  | Non-Completers (n=348) | 3.81 (0.66) |  |  |  |  |
| PHQ-4 | Completers (n=53) | 6.23 (3.4) | –3.88 | 58.33 | <.001 | 2.31 |
|  | Non-Completers (n=316) | 8.14 (2.05) |  |  |  |  |
| PHQ-D | Completers (n=53) | 10.47 (3.9) | –4.18 | 61 | <.001 | 3.0 |
|  | Non-Completers (n=316) | 12.81 (2.83) |  |  |  |  |
| WAI-SR | Completers (n=53) | 3.19 (0.90) | –5.15 | 61 | <.001 | 0.67 |
|  | Non-Completers (n=316) | 3.88 (0.60) |  |  |  |  |
| Mini-SPIN | Completers (n=53) | 7.73 (3.29) | –2.37 | 59 | <.001 | 2.3 |
|  | Non-Completers (n=316) | 8.87 (2.17) |  |  |  |  |

# Appendix E.

**Interaction with Clare®**

**Table 1** shows the mean and median number of calls in week 1-4 for the midtreatment

Sample after 4 weeks with Clare® (completed questionnaires at baseline, t1b, and t2; see

Fig. 1).

**Table 2** shows the mean and median average call length in seconds in week 1

4 for the midtreatment sample after 4 weeks with Clare® (completed questionnaires at

baseline, t1b, and t2; see Fig. 2). All participants included had at least one call with Clare®.

**Table 3** shows the mean and median number of calls in week 1-8 for the posttreatment

Sample after 8 weeks with Clare® (completed questionnaires at baseline, t1b, t2 and t3;

see Fig. 3).

**Table 4** shows the mean and median average call length in seconds in week 1-8 for the

posttreatment sample after 8 weeks with Clare® (completed questionnaires at baseline,

t1b, t2, and t3; see Fig. 4 in the manuscript). All participants included hat at least three

calls with Clare®, with minimum one call happening in week 1-4 and 1-8.

**Table E.1**

*Number of calls in week 1-4 in midtreatment-sample (n=53; t2)*

| **week** | **Mean (SD)** | **median** |
| --- | --- | --- |
| 1 | 1.77 (1.52) | 1.0 |
| 2 | 0.68 (0.96) | 0.0 |
| 3 | 0.49 (0.64) | 0.0 |
| 4 | 0.40 (0.57) | 0.0 |

**Table E.2**

*Average call length in week 1-4 in midtreatment-sample (n=53; t2)*

| **week** | **Mean (SD)** | **median** |
| --- | --- | --- |
| 1 | 201.17 (262.25) | 46.0 |
| 2 | 120.83 (193.59) | 0.0 |
| 3 | 74.89 (152.83) | 0.0 |
| 4 | 87.08 (176.72) | 0.0 |

**Table E.3**

*Average call length in week 1-8 in posttreatment-sample (n=21; t3)*

| **week** | **Mean (SD)** | **median** |
| --- | --- | --- |
| 1 | 237.52 (305.96) | 102.0 |
| 2 | 194.91 (204.12) | 136.0 |
| 3 | 144.29 (216.17) | 29.0 |
| 4 | 167.62 (223.29) | 45.0 |
| 5 | 112.05 (177.38) | 11.0 |
| 6 | 109.86 (210.59) | 0.0 |
| 7 | 56.00 (185.99) | 0.0 |
| 8 | 174.24 (260.64) | 30.0 |

**Table E.4**

*Number of calls in week 1-8 in posttreatment-sample (n=21; t3)*

| **week** | **Mean (SD)** | **median** |
| --- | --- | --- |
| 1 | 2.1 (1.48) | 2.0 |
| 2 | 0.95 (0.92) | 1.0 |
| 3 | 0.76 (0.77) | 1.0 |
| 4 | 0.62 (0.59) | 1.0 |
| 5 | 0.76 (0.70) | 1.0 |
| 6 | 0.52 (0.75) | 0.0 |
| 7 | 0.52 (0.75) | 0.0 |
| 8 | 0.57 (0.51) | 1.0 |
